# Evolved computers with culture. Commentary: From computers to cultivation: reconceptualizing evolutionary psychology

**DOI:** 10.3389/fpsyg.2015.00498

**Published:** 2015-04-22

**Authors:** Gregory A. Bryant

**Affiliations:** Department of Communication, University of California, Los AngelesLos Angeles, CA, USA

**Keywords:** computational theory of mind, domain specificity, evolutionary psychology, cultural learning, e-cognition

Barrett et al. review recent developments in evolutionary psychology (EP) and conclude that EP offers little in way of theoretical advancement over standard computational theory of mind (CTM) accounts, because traditional approaches in psychology implicitly accept that cognition is evolved. To Barrett et al., historical resistance to EP is surprising given that EP assumes the traditional computational-representational model of cognition. Across cognitive psychology, however, evolutionary approaches are sometimes accepted, but often mostly ignored. Vision and auditory perception researchers are typically functionalist, and as a result have made advances exceeding other areas of cognitive science—these scholars are often friendly to EP at least in some form. But many other areas have neither adopted a functionalist perspective nor currently accept the research program of EP. Certainly, most researchers in cognitive psychology do not attempt to reverse engineer computational solutions to adaptive problems as EP does. Not coincidentally, many cognitive psychologists study what EP would consider nonfunctional by products.

Still, it is true that EP has largely embraced cognitive psychology (though actual cognitive research is still surprisingly rare) and has integrated it with theories from evolutionary biology. Barrett et al. suggest that the adoption of the CTM constitutes a weakness for EP and they instead propose that various forms of e-cognition (i.e., embodied, embedded, enactive) offer a viable alternative to computational approaches. But as Klasios (2014) pointed out in his recent commentary, Barrett et al. fail to recognize that “cognitive integration” is information processing, and in its most basic sense, is necessarily computational. As Gallistel and King (2009) recently put it, describing the mind as a case of digital computation “is the only game in town” (p. 24). There is no scientific alternative to the notion that the neural coding of events in the world involves the probabilistic transformation of information. If one admits that much, logical entailments prevent the kind of rejection of the CTM that Barrett et al. endorse.

Notions of e-cognition can be provocative, and on the surface can seem like an advancement in our ideas about human cognition. However, there are some fundamental problems in the current presentation and the ideas in general. By suggesting that our cognition is shaped by cultural artifact use, I believe Barrett et al. point the causal arrow mostly backwards. That is not to say that artifacts cannot, in principle, affect brain organization, but the evidence to date seems to favor the idea that cultural phenomena are generally tailored to our brains and bodies, not the reverse (Claidiere and Sperber, 2007). For instance, the authors use the example of time-pieces contributing to culturally evolved values associated with timeliness, and they attribute timeliness as being part of our human nature, essentially arguing that extended artifacts like time-pieces have altered our cultural cognitive machinery. There is no question that inventions like time-pieces feed back into practices and beliefs—the more we advance the technology, the more we allow ourselves to be manipulated by it. But timeliness is a byproduct of social coordination, cooperation, and reciprocity. If an individual demands that her associate pay attention to the time—an ability afforded by a time-piece—and then the associate does not abide when able to do so, he is implicitly discounting the value of the relationship. The human nature component in this example is not the timeliness per se, but the use of culturally evolved norms as a means to coordinate social interactions.

Admittedly, the ways neural coding schemes relate to various phenomena in the world, external to the brain itself, constitute hard empirical questions that will almost certainly need to incorporate many complexities suggested by various forms of e-cognition. These issues will likely be resolved, however, within a framework that involves, at its theoretical core, computational mechanisms implemented in the brain. Even if some of the external phenomena that e-cognition proponents describe constituted legitimate examples of extended phenotypic traits (Dawkins, 1982), their implementation would still land squarely in the neural circuitry of the brain interfacing with motor systems. For example, written language is learned by people quite effectively and writing systems are shaped by both cultural and cognitive factors, including visual processing and memory systems. There is evidence of a brain area that, when given certain input, reliably develops expertise for visual words (Dehaene, 2009), showing amazing flexibility in how brain structure interacts with culture (Barrett, 2012). But our understanding of the psychology of reading is purely computational. Similarly, we don't need a special theory of beaver cognition because of beaver dams—we just need to explain the evolved cognitive and behavioral processes that allow beavers to build them.

Evolutionary behavioral scientists who study culture often rely on the concept of domain generality, presumably because cultural phenomena seemingly incorporate so many aspects of our cognition and environment. Of course, culture is deeply interconnected with many facets of our cognitive processing, but that does not require a system that is infinitely flexible and unconstrained by past selection. Rather, culture is rooted in a suite of cognitive and communicative abilities that allow us to transmit rich information vertically and horizontally, and the outputs of such processes feedback iteratively into an evolutionarily dynamic cultural knowledge system rooted in adaptive computational design. Cultural transmission often follows certain patterns resulting in stable psychological and communicative strategies that have all the hallmarks of domain specificity: (i) our attention is directed in specific ways to particular relevant agents, (ii) motivational systems drive the spreading of specific kinds of information, and iii) cultural learning systems are content sensitive.

The authors acknowledge the idea that there is no defensible dividing line between domain specific and domain general mechanisms (Barrett and Kurzban, 2006), but then they fail to properly appreciate this in their treatment of certain culturally learned information, such as the special status of incest taboos in cultural transmission. In the example given, Barrett et al. fail to acknowledge the possibility that unconscious processes guiding incest avoidance (Lieberman et al., 2007) were driving the mating decisions described by Durham (2002) despite variations over time in the local cultural rules. Overall, they emphasize examples of domain-general mechanisms potentially solving problems that some evolutionary psychologists consider only manageable by highly specialized domain-specific systems—but seem to momentarily forget that just because a mechanism works across content domains, it is still functionally specialized. The scope of a mechanism is independent from whether it has design features (i.e., functional specialization) (Barrett and Kurzban, 2006). Cognitive mechanisms, including associative learning processes and various decision making systems sensitive to local information, can operate on representations across multiple domains and subsequently feed into more specialized systems—cognition is hierarchically structured, and evolutionarily conserved (Barrett, 2012). So where is the argument exactly?

Despite these disagreements—some apparent, some real—Barrett et al. seem to illustrate that the historical gap between behavioral ecology and evolutionary psychology is closing, not widening. Many evolutionary psychologists are developing a greater appreciation for cultural evolution and behavioral flexibility, and behavioral ecologists are more concerned now with cognitive adaptations and experimental psychology methodology. Both fields have led the behavioral sciences in cross-cultural fieldwork, and to a great extent, we share a theoretical foundation. Don Symons's question (1987) still looms, however: If we're all Darwinians, what's the fuss about?

## Conflict of interest statement

The author declares that the research was conducted in the absence of any commercial or financial relationships that could be construed as a potential conflict of interest.
